# Influence of Different Types of Surfactants on the Flotation of Natural Quartz by Dodecylamine

**DOI:** 10.3390/molecules29102256

**Published:** 2024-05-11

**Authors:** Yuxin Ao, Cong Han, Linghao Kong, Yanbai Shen, Sikai Zhao, Wengang Liu, Shijie Zhou

**Affiliations:** 1 School of Recourse and Civil Engineering, Northeastern University, Shenyang 110819, China; aoyuxin@gmail.com (Y.A.); linghaokong799@outlook.com (L.K.); zhaosikai@mail.neu.edu.cn (S.Z.); liuwengang@mail.neu.edu.cn (W.L.); zhoushijie@mail.neu.edu.cn (S.Z.); 2 State Key Laboratory of Process Automation in Mining & Metallurgy, Beijing 100160, China

**Keywords:** surfactant, dodecylamine, quartz, flotation, foam

## Abstract

The synergistic effect among flotation agents is why combined flotation agents exhibit superior performance compared to single flotation agents. This research investigates the influence of three surfactants with different charges of polar groups, sodium dodecyl sulfate (SDS), cetyltrimethylammonium bromide (CTAB), and octanol, combined with dodecylamine (DDA), on quartz flotation. Through the implementation of flotation tests, bubble–particle adhesion induction time testing, gas–liquid two-phase foam properties testing, and surface tension testing, it is revealed that substituting part of the DDA with these surfactants can either enhance or at least maintain the quartz recovery, affect the adhesion induction time, reduce the surface tension of the flotation system, and change the foaming performance and foam stability, depending on their mole ratio in the combined collector. Compared to DDA alone, combining CTAB or OCT with DDA can significantly increase quartz recovery, while SDS with DDA only yields an approximate recovery. Combining SDS or OCT with DDA can reduce the foam stability, while CTAB with DDA enhances the foam stability. The effect of the combination of surfactants and DDA on the adhesion induction time of quartz grains of different sizes with bubbles is the same; furthermore, there is a negative correlation between the adhesion induction time and the recovery, while the foaming properties and stability of foam are positively correlated with the recovery.

## 1. Introduction

Quartz is a typical silicate mineral and is common gangue mineral in the beneficiation process of various types of ores [1,2,3]. Flotation is one of the main methods to separate quartz from ores [4,5]. Taking iron, a strategic metal mineral, as an example, efficiently removing silicate minerals, including quartz, from iron ore is one of the critical objectives in iron ore beneficiation. Using flotation to separate quartz from ore currently involves two technologies: anionic collector flotation and cationic collector flotation, which have been widely applied in industrial production [6,7]. Anionic reverse flotation processes have the advantages of easy separation of gangue minerals and relatively high selectivity of reagents. Therefore, anionic reverse flotation processes hold a significant share in applying iron ore flotation for desilication. However, anionic reverse flotation still requires improvement due to various reagents, high reagent dosages required, and complex reagent applying conditions. Cationic reverse flotation for desilication has the advantages of simple reagent systems, low reagent dosage, easy operation, and so on. However, high foam viscosity, flotation foam prone to carrying gangue minerals, poor selectivity, and other inadequacies limit the wide use of the cationic reverse flotation process. As a result, its proportion in industrial production is still lower than that of the anionic reverse flotation process. Many researchers have been exploring combining and modifying commonly used cationic collectors to improve the shortcomings of cationic reverse flotation processes.

The most widely used cationic collectors currently include DDA, quaternary ammonium salts, ether amines, CS series, GE series, etc. [8,9,10,11,12], among which DDA is the most representative. Many researchers have conducted extensive research on the flotation application of DDA to improve its flotation performance. They have found that many different reagents can synergize with DDA and obtain better flotation indicators. Wang et al. found that when using a combined collector of sodium oleate and DDA for the flotation of scheelite and calcite, the recovery was higher compared to using DDA alone [13]. Luo et al. investigated the influence of BHA/DDA on the selective separation of ilmenite and titaniferous diopside. They found that the BHA-DDA complex adsorbed on the surface of ilmenite through chemical adsorption of BHA and electrostatic adsorption of DDA. In contrast, the complex exhibited unstable adsorption on titaniferous diopside [14].

In foam flotation, hydrophobic mineral particles adhere to bubbles to float, thereby separating from hydrophilic minerals. The interaction between bubbles and particles can usually be divided into three stages: collision, adhesion, and desorption, which are the critical processes in achieving flotation separation. They are affected by bubble surface characteristics and particle and solution chemical properties [15]. Induction time is an essential parameter described in bubble–particle adhesion, which is the time required for liquid film drainage, liquid film rupture, formation, and expansion of the three-phase contact line between bubbles and particles [16]. In the flotation process, induction time and contact angle are frequently used to evaluate floatability and flotation recovery [17]. Generally speaking, the contact angle is measured in an equilibrium state, while the induction time is measured dynamically. Therefore, the latter is more suitable for describing mineral flotation behavior for the flotation system. For example, in a coal flotation system, contact angle has been proven to be unable to explain the flotation behavior of coal particles [18,19], while induction time can better characterize the flotation effect [20,21].

Amine-type cationic collectors act as surfactants and also have foaming properties in flotation. This means that when other flotation reagents, especially surfactants and DDA, are combined and used for flotation, the system’s foaming property, the mineral’s wettability, and the flotation foam’s properties will be affected, suggesting that it is possible to control the adhesion between bubbles and particles and the properties of flotation foam by using a combined collector, thus improving the flotation performance of cationic collectors. The flotation foam performance of amine collectors currently used in cationic reverse flotation desilication processes for iron ore still cannot fully meet production requirements. Therefore, improving the flotation foam performance of amine collectors is also one of the hot research topics in the application of cationic reverse flotation desilication technology for iron ore. Liu et al. demonstrated through surface tension experiments that introducing varying amounts of isopropanol substituents into DDA can enhance its hydrophobicity and reduce the amount of reagent required [22]. Qiao Xiaoxiao found through experiments that the addition of iso-octanol can improve the foaming performance of dodecylamine, making the foam more brittle and readily dispersible [23]. The existing research results show that different types of surfactants can have synergistic effects with DDA, thus changing the system’s interfacial tension and flotation foam performance and then affecting the flotation process. However, the differences and characteristics of synergistic effects between different types of surfactants and DDA and the relationship between the flotation process and the flotation effect still need to be completed.

In our previous work, we examined the influence of OCT, SDS, and CTAB surfactants on the flotation of quartz by DDA. It is found that all three surfactants can co-adsorb with DDA on the surface of the quartz, thus strengthening the hydrophobicity of the quartz surface and promoting quartz floatability. Due to the different electrical properties of polar groups, the thickness and stability of the adsorption layer formed by the co-adsorption of three surfactants with DDA on the quartz surface and the influence on the zeta potential of the quartz surface are different [24]. However, the previous studies were mainly carried out from the perspective of changing mineral surface properties and did not involve gas–liquid interface properties, bubble–particle adhesion, or foam properties. According to the literature reports, the surface tension will significantly impact the adhesion between bubbles and particles and the performance of flotation foam [21,25]. Therefore, this study still selects OCT, SDS, and CTAB as before and combines them with DDA, focusing on their synergistic effects on the surface tension of the flotation system, the induction time of bubble–particle adhesion, and the flotation foam performance to deeply understand the influence mechanism of surfactants on cationic collector flotation of quartz and enrich the theoretical research on the synergistic effect of combined flotation reagents.

## 2. Results and Discussion

### 2.1. DDA Flotation Performance

**Figure 1:**
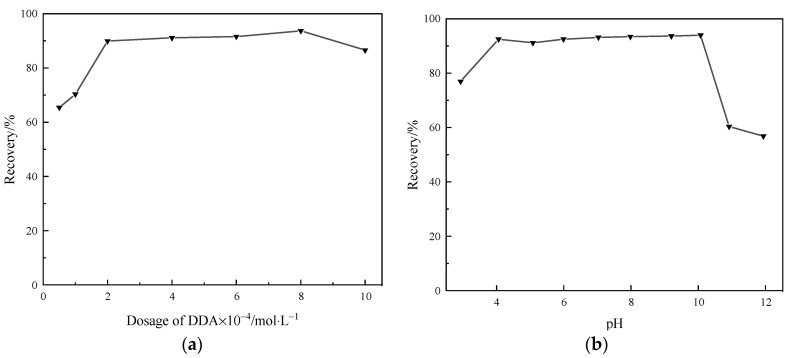
Effect of (**a**) dosage of DDA and (**b**) pH on the quartz recovery (%).

A series of flotation tests were conducted separately to investigate the effect of DDA dosage and slurry pH on the floatability of quartz. The pH was maintained at 10.1 while examining the effect of collector dosage, and the DDA dosage was set at 8 × 10^−4^ mol/L while investigating the impact of pH. The results are illustrated in Figure 1.

It can be observed from Figure 1a that with the increase in DDA dosage, the recovery of quartz initially increases rapidly, then the rate of increase gradually decreases and finally stabilizes. When the DDA concentration ranges from 2 × 10^−4^ mol/L to 8 × 10^−4^ mol/L, the recovery of quartz remains relatively stable. When the DDA concentration reaches 8 × 10^−4^ mol/L, the maximum recovery of quartz is 93.71%. Beyond this concentration, as DDA concentration continues to increase, the recovery of quartz gradually decreases.

As shown in Figure 1b, with the increase in slurry pH, the recovery of quartz initially increases and then decreases. As the slurry pH rises from 3.0 to 10.1, the recovery of quartz gradually increases from 76.96% to 93.71%. Beyond this pH, as the slurry pH continues to rise, the recovery of quartz gradually decreases. When the slurry pH rises to 11.9, the recovery of quartz decreases to 56.83%.

### 2.2. Combined Collector Flotation Performance

**Figure 2:**
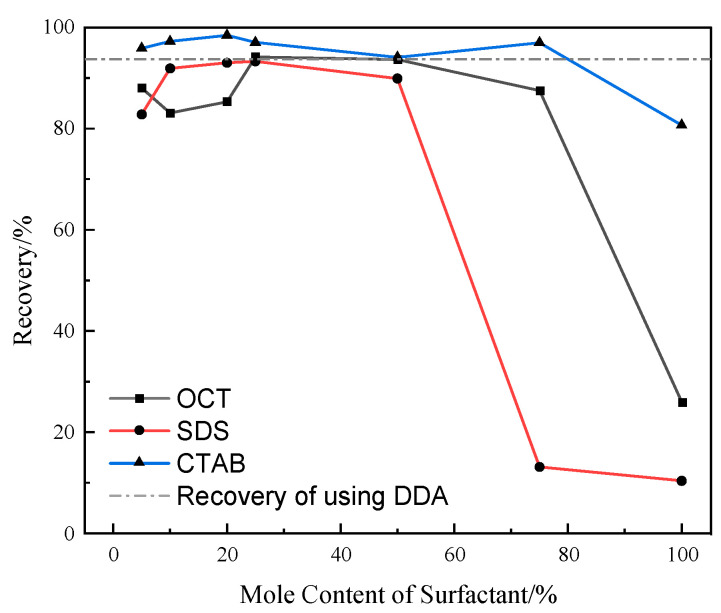
Effect of surfactant mole content in combination collectors on quartz recovery (%).

The influence of the molar content of surfactants on the recovery of quartz was investigated at a slurry pH of 10.1 and a combined collector concentration of 8 × 10^−4^ mol/L, and the results are shown in Figure 2.

Figure 2 shows that while the molar content of OCT is less than 25%, the recovery of quartz exhibits a trend of first decreasing and then increasing with the increase in OCT molar content. The maximum recovery is 94.15%, slightly higher than using DDA alone. When the molar content of OCT ranges from 25% to 75%, the recovery of quartz gradually decreases with the increase in OCT content. However, when the molar content of OCT exceeds 75%, the recovery of quartz starts to decline rapidly. When the molar content of OCT reaches 100%, the recovery of quartz is 25.98%. As OCT has no collecting effect on quartz [24], the quartz entering the foam is mainly due to the mechanical entrainment of the foam during flotation.

When the molar content of SDS is less than 25%, the recovery of quartz shows a gradual increase with the increase in SDS molar content. The maximum recovery is 93.30%, slightly lower than when using DDA alone. However, while the molar content of SDS exceeds 25%, the recovery of quartz gradually decreases. While the molar content of SDS reaches 100%, the recovery of quartz is 10.38%. Similar to OCT, as SDS has no collecting effect on quartz, the quartz entering the foam is mainly due to the mechanical entrainment of the foam during flotation.

When the molar content of CTAB is less than 50%, the recovery of quartz shows a trend of first increasing and then decreasing. When the molar content of CTAB is 20%, the maximum recovery is 98.43%, higher than the recovery while using DDA alone. However, when the molar content of CTAB exceeds 50%, the recovery of quartz shows a trend of first increasing and then decreasing. When the molar content of CTAB is 100%, the recovery of quartz is 80.68%.

The above results show that when the dosage of collectors remains constant, the three types of surfactants can partially replace DDA in the combined collectors within a specific molar ratio range and achieve recovery similar to that obtained when using DDA alone for quartz flotation. One reason for this phenomenon is that the three types of surfactants can undergo co-adsorption with DDA on the surface of the quartz, enhancing the hydrophobicity of the quartz surface [24]. Another possible reason is that the three types of surfactants can undergo co-adsorption with DDA at the gas/liquid interface, altering the properties of the original gas/liquid interface in the system. This change affects the adhesion behavior between quartz particles and bubbles, as well as the properties of the flotation foam, thereby influencing the recovery of quartz.

### 2.3. Induction Time of Bubble–Particle Adhesion

From collision to adhesion, mineral particles and bubbles must undergo four processes: thinning and rupturing bubble hydration film, expansion of the three-phase wetting periphery, and particles’ stable adhesion to bubbles. The total time of the four stages is known as the induction time of bubble–particle adhesion, abbreviating induction time, which is related to properties such as particle size, surface wettability, and interfacial tension between gas and liquid [16,26,27,28]. Generally speaking, the stronger the hydrophobicity of the mineral surface, the shorter the induction time, which means that bubbles and particles are more straightforward to adhere to and the greater the flotation recovery. The time from collision to desorption of particles is called the contact time, so to adhere to bubbles successfully, the induction time must be less than the contact time [29]. Introducing new surfactants can change the original surface tension of the DDA system, thus affecting bubble hydration film thinning, bubble rupture, and three-phase wetting peripheral expansion during the adhesion process between particles and bubbles, hence changing the adhesion behavior between bubbles and particles [30].

**Figure 3:**
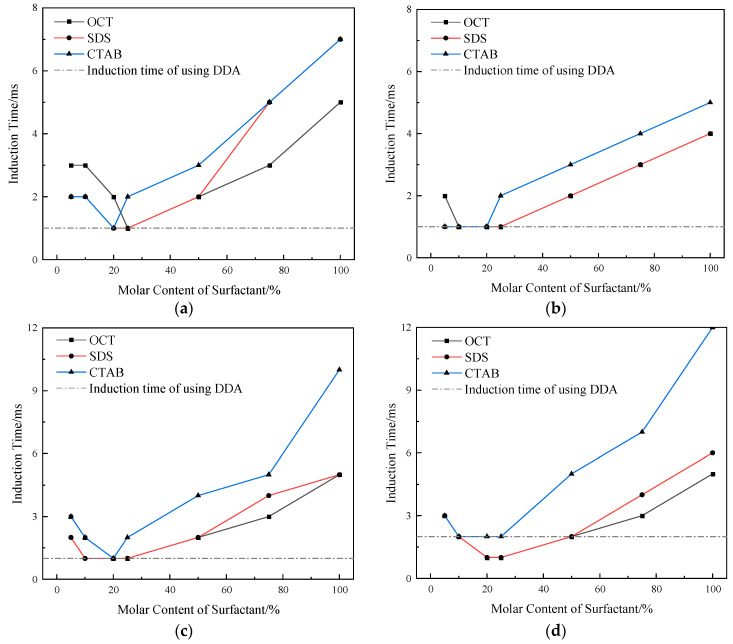
Effect of surfactant mole content of in combination collector on induction time of particles of different particle sizes (**a**) 74~100 μm; (**b**) 54~74 μm; (**c**) 38~54 μm; (**d**) 22~38 μm.

The influence of the molar content of surfactants in the combined collectors on the induction time was investigated at a slurry pH of 10.1 and a combined collector concentration of 8 × 10^−4^ mol/L, and the results are shown in Figure 3.

As seen in Figure 3, the induction time tends to decrease first and then increase with the increase in the molar content of the three surfactants for the same particle size and flotation system. The induction time is the smallest when OCT, SDS, and CTAB account for 25%, 25%, and 20% of the molar content of the combined collector, respectively, and it is similar to that when using DDA alone. When the particle size and the surfactant molar content are the same, the order of value of the induced time is CTAB > SDS > OCT in the combined collector system of three surfactants and DDA, respectively. When the molar content of three surfactants is the same, the induced time tends to decrease first and then increase with the decrease in particle size. As the particle size is between 54 and 100 μm, the induced time tends to decrease; the induced time of 54~74 μm is the smallest, and the induced time increases when the particle size is less than 54 μm, among which the CTAB system increases the most. The reason the induction time of coarse particles is longer is that they need greater adhesion to overcome their gravity and adhere to bubbles [31], while the reason why the induction time of fine particles increases may be because of their small mass and small kinetic energy, which makes it difficult to break the hydration layer after collision with bubbles completely.

### 2.4. System Surface Tension

**Figure 4:**
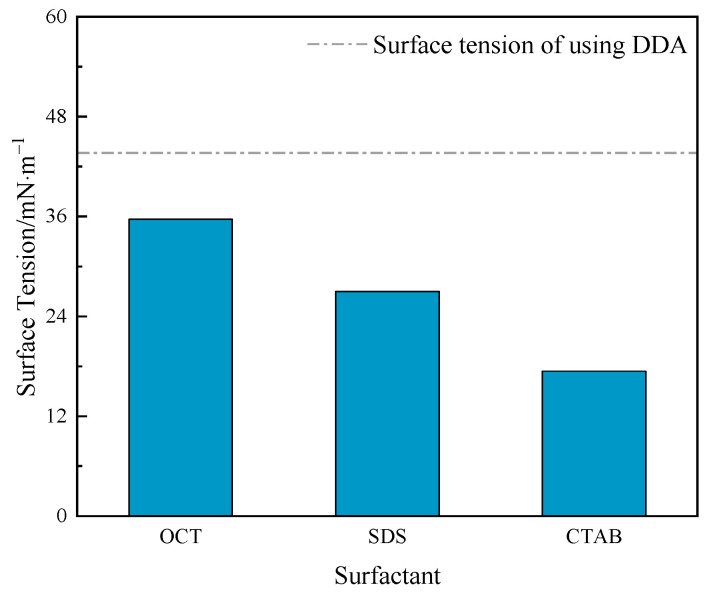
Effect of surfactants on the surface tension of the system.

Reducing the surface tension of the flotation system is necessary for generating and maintaining stable foam. Additionally, the more significant the decrease in surface tension, the stronger the surfactant activity of the reagent, implying lower energy required for bubble generation [32]. Therefore, with the same amount of reagents, the stronger the surface activity, the more bubbles are generated, and the greater the probability of collision and adhesion between mineral particles and bubbles, thus achieving higher flotation recovery [33]. For amine collectors, combined reagents with other surfactants usually exhibit higher surface activity [34]. To investigate the effect of the combination of surfactants on the surface tension of the system, surface tension measurements were conducted under the conditions of pH 10.1, collector concentration of 8 × 10^−4^ mol/L, and molar contents of OCT, SDS, and CTAB at 25%, 25%, and 20%, respectively. The results are shown in Figure 4.

As shown in Figure 4, the surface tension of the combined reagent system is lower than that of the lonely DDA system. It indicates that the co-adsorption of the three surfactants with DDA at the gas/liquid interface has reduced the system’s surface tension. The order of decrease in the system’s surface tension is CTAB > SDS > OCT.

### 2.5. Properties of Two-Phase Foam

Two-phase foam is a kind of foam that only contains a gas and liquid phase but not a solid phase [25]. The maximum foam volume and half-life are important indicators for evaluating foam performance. The maximum foam layer volume represents the amount of foam produced by the system, indicating the foaming performance of the reagent. The half-life suggests the ability of the foam to maintain its original state, reflecting the stability of the flotation foam [35]. The maximum foam volume determines whether flotation separation can be processed; only when a specific volume of the foam layer is generated can flotation separation be achieved. The stability of foam has a significant impact on flotation indicators. Unstable foam can cause separated mineral particles to re-enter the slurry phase, reducing flotation efficiency and concentrate yield. On the other hand, excessively stable foam is difficult to break, making it challenging for hydrophilic gangue minerals mechanically entrapped in the foam to re-enter the slurry, thus reducing concentrate grade. Additionally, excessively stable foam hinders froth scraping operations and may lead to overflow issues during production, adversely affecting the generation process [36].

**Figure 5:**
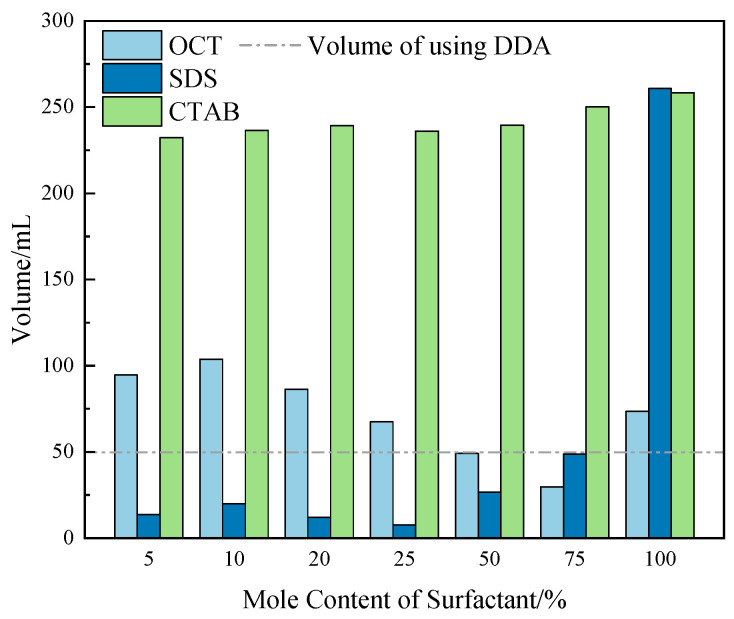
Effect of surfactant mole content in combined collector on foam volume.

**Figure 6:**
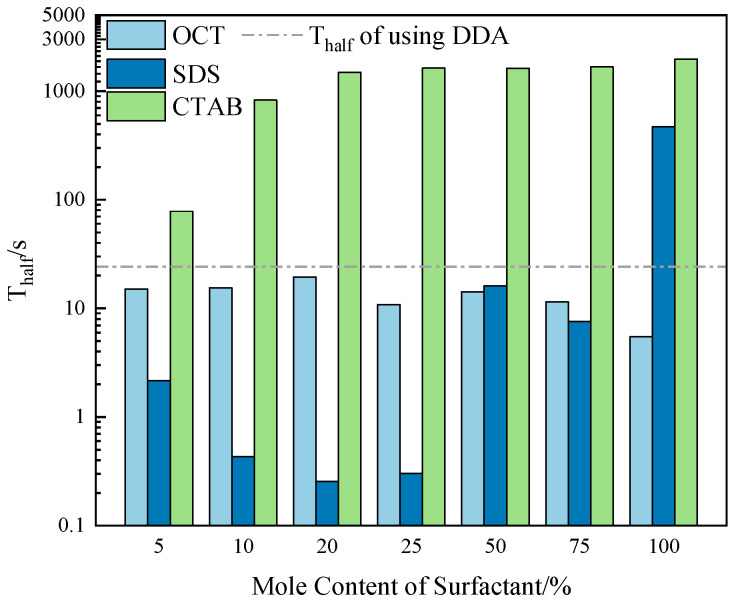
Effect of surfactant mole content in combined collector on foam half-life.

To investigate the effect of three surfactants on the foam properties of the DDA system at a collector concentration of 1 × 10^−3^ mol/L, a pH value of 10.1, an air flow rate of 0.6 L/min, and an aeration time of 30 s, the effect of the molar content of surfactants in the combined collector on foam layer volume and foam stability (half-life) was examined. The results are shown in Figure 5 and Figure 6.

#### 2.5.1. Foaming Properties

Figure 5 shows that when the concentrations are all 1 × 10^−3^ mol/L, the foaming properties of the three surfactants are more potent than those of DDA. Among them, the foaming properties of OCT and DDA are similar and much weaker than those of SDS and CTAB. However, due to the limitations of the dynamic foam analyzer range (260 mL), the experiment automatically stops when the generated foam reaches the overflow warning line, so it is currently impossible to determine the relative foaming properties of SDS and CTAB at the current concentrations. The interaction between the three surfactants and DDA has a significant difference in the foaming properties of the combined collector. In the OCT/DDA system, when the molar content of OCT is less than 75%, the maximum foam layer volume of this system shows a trend of first increasing and then decreasing. The volume is maximized when the molar content of OCT is 10% and minimized when it is 75%, even lower than that of the DDA system. When the molar content of OCT is greater than 75%, the maximum foam layer volume gradually increases. In the SDS/DDA system, when the molar content of SDS is less than 25%, the maximum foam layer volume of this system shows a trend of first increasing and then decreasing, but overall fluctuates little. When the molar content of SDS is greater than 25%, the maximum foam layer volume gradually increases. When the molar content of SDS is less than 75%, the foaming properties of the SDS/DDA system are lower than those of the DDA system. Regarding the CTAB/DDA combination, the foaming properties are much stronger than those of the DDA system, and the molar content of CTAB has little influence on the foaming properties of the combined collector system.

From an overall perspective, when the molar content of surfactants in the combined collector is less than 50%, compared to the foaming properties of DDA, CTAB, and OCT combined with DDA, it can strengthen the foaming properties of the system, while SDS combined with DDA reduces the foaming properties of the system.

#### 2.5.2. Foam Stability

Figure 6 shows that the interaction between the three surfactants and DDA significantly differs in the foam’s stability generated by the combined collector system. In the OCT/DDA system, with the increase in OCT molar content, the stability of the generated foam fluctuates to some extent. Still, it shows a decreasing trend overall and is close to the stability of the foam generated by the DDA system. In the SDS/DDA system, when the SDS molar content is less than 75%, the foaming properties of the SDS/DDA system are lower than those of the DDA system. With the increase in SDS molar content, the stability of the generated foam shows a rapid decrease, followed by stabilization. When the SDS molar content is between 20% and 25%, the stability of the generated foam remains relatively constant. However, when the SDS molar content exceeds 25%, the stability of the generated foam rapidly increases, and the maximum value is higher than that of the stability of the foam generated by the DDA system by an order of magnitude. In the CTAB/DDA system, when the CTAB molar content is less than 5%, the stability of the generated foam shows a rapid increase with the increase in CTAB molar content. When the CTAB molar content exceeds 20%, the stability of the generated foam remains relatively constant.

Overall, when the molar content of SDS and OCT in the combined collector is less than 75%, compared to the stability of foam generated by the DDA system, the stability of foam generated by the combined SDS and OCT with DDA can be reduced, while the combination of CTAB and DDA enhances the stability of the generated foam. Additionally, at a concentration of 1 × 10^−3^ mol/L, CTAB exhibits the highest foam stability, followed by SDS, and OCT has the lowest foam stability. Specifically, the stability of foam generated by CTAB is approximately 100 times that of foam generated by DDA, the stability of foam generated by SDS is approximately 50 times that of foam generated by DDA, and the stability of foam generated by OCT is less than that of foam generated by DDA but within the same order of magnitude.

#### 2.5.3. Structure of Generated Foam

**Figure 7:**
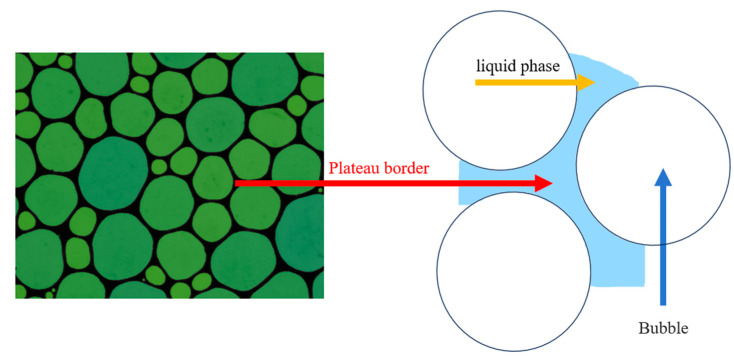
Schematic diagram of foam layer plateau channels.

Foam is a dynamic system composed of three structural elements: liquid films, nodes, and Plateau channels, following Plateau’s law. In the equilibrium state, adjacent bubbles in the foam are separated by liquid films, forming a three-forked structure with a 120° angle between the adjacent liquid films, namely the Plateau channels, as shown in Figure 7. The fluid film in the Plateau channel is one basic structural unit of the foam. The liquid film’s thickness directly affects the foam’s drainage and stability. In contrast, the drainage performance of the foam is closely related to the secondary enrichment effect of flotation foam [37].

**Figure 8:**
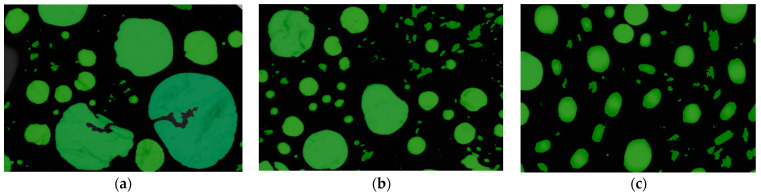
Snapshot of foam structures generated by different collector systems: (**a**) DDA; (**b**) OCT/DDA; (**c**) CTAB/DDA.

To examine the influence of surfactants on foam structure, at the conditions of collector concentration of 1 × 10^−3^ mol/L, pH of 10.1, and aeration flow rate of 0.6 L/min with an aeration time of 30 s, the structures of foam generated by a single DDA collector, a combination of OCT/DDA with a 25% mole content of OCT, and a combination of CTAB/DDA with a 20% mole content of CTAB at the 15th second of aeration were recorded. The snapshots of the foam structures are shown in Figure 8.

Figure 8 and Table 1 show that under the same conditions, the DDA collector system generates the fewest bubbles and the largest average bubble area, but with an uneven bubble area. Compared to the single DDA system, the system using OCT in combination with DDA shows a significant increase in the number of generated bubbles, a decrease in the average area of the bubble, and an increase in the homogeneity of the bubble area. Similarly, the system using CTAB in combination with DDA generates even more bubbles than the OCT/DDA system, with a smaller average bubble area and a more uniform distribution of bubble area.

It has been known that the liquid drainage from the fluid film mainly consists of two parts: gravity drainage within the Plateau channels and thermodynamic drainage within the fluid film. Gravity drainage is dominant when the fluid content inside the foam is high. As the thickness of the liquid film gradually decreases, thermodynamic drainage will become dominant [38].

According to the Laplace formula shown in Equation (1):

(1) $$\Delta p=\gamma \left(\frac{1}{R_{1}}+\frac{1}{R_{2}}\right)$$

where Δ*p* represents the pressure difference across the curved interface, *γ* is the surface tension coefficient, and *R*_1_ and *R*_2_ are the radii of curvature of the liquid interface.

When the surface tension of the system decreases, the pressure difference across the liquid interface decreases, leading to a slower drainage rate of the foam liquid film through the Plateau channels and enhancing the stability of the foam. Under experimental conditions, the CTAB/DDA system exhibits the lowest surface tension, thus having the lowest drainage capacity for foam formation. Consequently, the stability of the foam generated by the CTAB/DDA system should be greater than that generated by the single DDA and OCT/DDA systems, consistent with the results of foam stability tests. Furthermore, more robust foam stability leads to slower foam drainage rates, excellent resistance of foam bubbles to deformation, and more difficulty colliding mineral particles in foam to break the hydration layer of bubbles, which increases the induction time and is consistent with the results of induction time detection. On the other hand, as the surface tension of the flotation system is closely related to its foaming properties, reducing surface tension within a specific range promotes foam generation in the flotation system. Therefore, it can be inferred that the CTAB/DDA system will produce more bubbles under the same energy input conditions. Additionally, due to the slow drainage rate of Plateau channels within the foam and its strong stability, the foam layer volume generated by the CTAB/DDA system is maximized, consistent with the results of foaming property tests.

## 3. Materials and Methods

### 3.1. Materials

**Figure 9:**
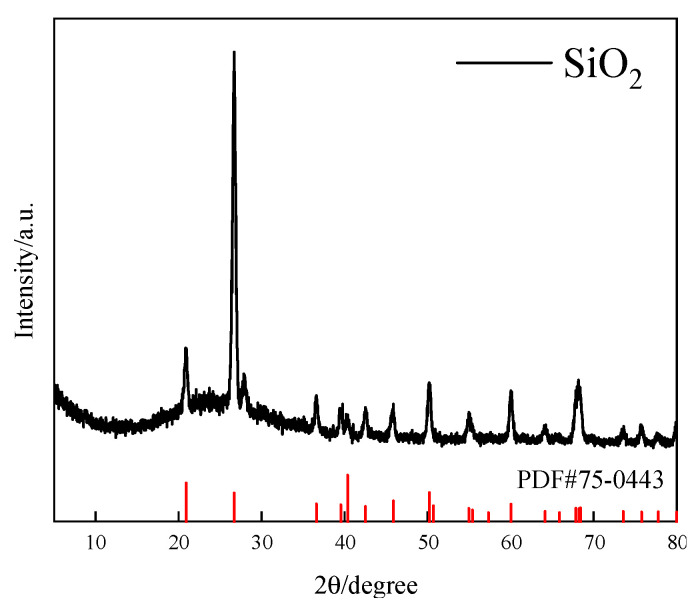
X-ray diffraction pattern of the sample.

**Figure 10:**
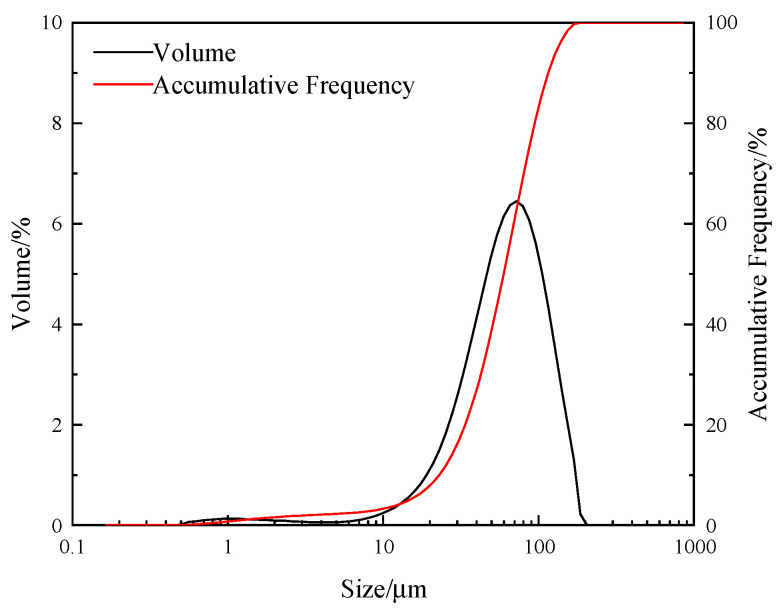
Particle size analysis results for the sample.

The quartz raw ore used in the experiment was obtained from a mine in Anshan, Liaoning Province, China. The ore samples were crushed, and high-purity, well-crystallized ore blocks were hand-selected. The hand-selected ore blocks were crushed, ground, and sieved to obtain products with a particle size of 15~74 μm. The sieved products were soaked in concentrated hydrochloric acid for 72 h, rinsed multiple times with deionized water, and dried in an oven at a low temperature (105 °C) to obtain samples for flotation experiments. The chemical multi-element analysis results of the flotation test samples are shown in Table 2. The samples were subjected to X-ray diffraction (XRD) analysis using a DISCOVER D8 diffractometer (Bruker, Karlsruhe, Germany), and the spectrum is shown in Figure 9. The particle size of the samples was analyzed using the Masterizer 2000 laser particle size analyzer (Malvern, Malvern, UK), and the analysis results are shown in Figure 10.

Table 2 shows that the SiO_2_ content in the sample is 99.57%, the Fe content is less than 0.05%, and there are also trace amounts of Al, K, and Ca elements. As shown in Figure 9, the diffraction peaks of the sample coincide with those of the standard quartz card, and no other prominent impurity diffraction peaks are present. Combining the results of the chemical multi-element analysis of the sample, it can be concluded that the sample is quartz with high purity, and the quartz content in the sample is 99.57%, which meets the requirements of single mineral flotation experiments.

As shown in Figure 10, it can be observed that the particle size distribution of quartz particles in the test sample is mainly concentrated in the range of 15~74 μm. Specifically, the content of particles in the 54~74 μm size range is 31.09%, in the 38~54 μm size range is 20.13%, in the 22~38 μm size range is 15.08%, and particles less than 22 μm constitute 8.54% of the sample. To investigate the relationship between quartz particle size and the collecting performance of the collector, further sieving of the prepared quartz sample was conducted using standard sieves with apertures of less than 54 μm, less than 38 μm, and less than 22 μm. The sieved products were collected for subsequent testing.

### 3.2. Reagents

Analytic-grade hydrochloric acid and sodium hydroxide as pH regulators were used for flotation experiments. The collector dodecylamine (DDA), surfactant octanol (OCT), sodium dodecyl sulfate (SDS), and cetyltrimethylammonium bromide (CTAB), as well as acetic acid, were all of chemical purity. All chemical reagents used in the experiments were purchased from the Sinopharm Chemical Reagent Co., Ltd. (Shenyang, China). During the experiments, dodecylamine and acetic acid were mixed in a 1:1 molar ratio and dissolved in deionized water to prepare the solution. In contrast, other chemical reagents were dissolved in deionized water to prepare the solution.

### 3.3. Methods

#### 3.3.1. Flotation Experiments

**Figure 11:**
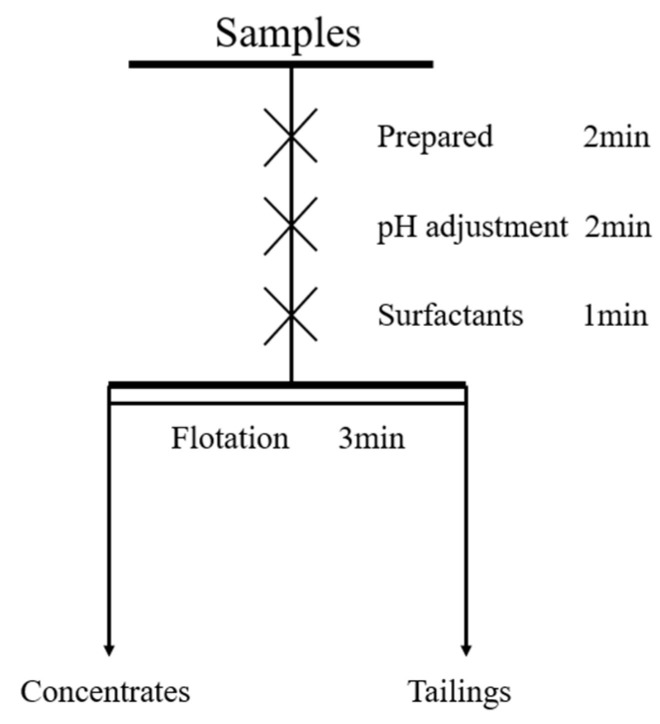
Flowsheet of single mineral flotation test.

The single mineral flotation experiments were conducted in an XFG hanging slot flotation machine (Jilin Exploration Machinery Plant, Changchun, China) with an impeller speed of 1992 r/min at room temperature. In each experiment, 2.0 g of ore sample was weighed and placed into a 30 mL flotation cell, followed by an appropriate amount of deionized water. After stirring for 2 min, the pH adjuster and collector were added sequentially, and the flotation process was initiated. During flotation, the foam was scraped every 10 s, with a total scraping time of 3 min. After the experiment, the foam product was dried and weighed, and the recovery was calculated using the difference method. The flotation experiment process is illustrated in Figure 11.

#### 3.3.2. Induction Time Measurements

**Figure 12:**
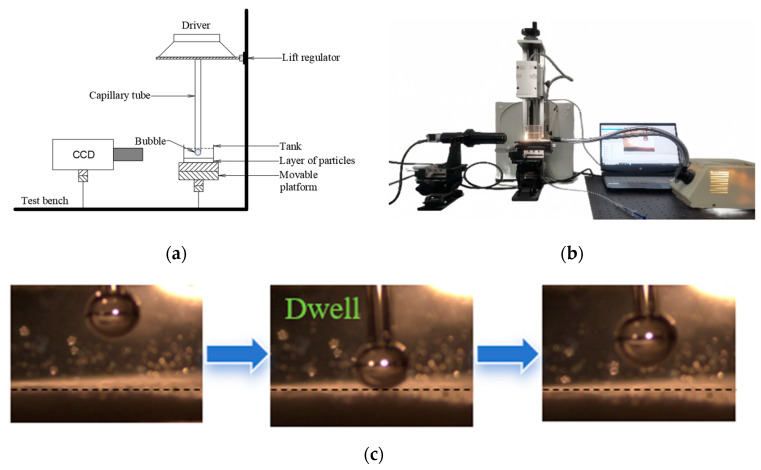
Bubble–particle adhesion induction time testing device and schematic diagram of the testing process. (**a**) Schematic diagram of device structure; (**b**) testing device; (**c**) process of testing bubble–particle adhesion induction time.

A self-constructed induction time measurement device determined the induction time for bubble–particle adhesion. The structure of the induction time measurement device and the testing process are illustrated in Figure 12. The induction time is the shortest contact time required for bubble and particle adhesion [39,40,41]. First, a 2.0 g ore sample is weighed and placed into the observation chamber. Then, an appropriate amount of deionized water is added to the observation chamber. Subsequently, the observation chamber is placed on a magnetic stirrer, and the stirring speed is set to 500 r/min. After stirring for 2 min, reagents are added according to the flotation test procedure, and the slurry is mixed. Finally, the observation chamber is placed on the observation platform for induction time measurement. During the induction time measurement process, five random points are selected on the particle bed layer, and each point is tested ten times repeatedly. The contact time at which adhesion occurs for 50% of the tests is recorded as the measured induction time.

#### 3.3.3. Foam Performance Test

**Figure 13:**
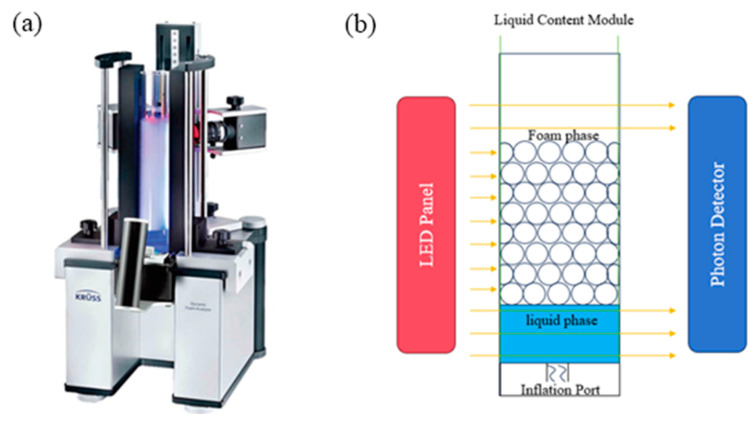
DFA-100 Dynamic Foam Analyzer (**a**); Detection principle schematic diagram of DFA-100 (**b**).

The content of two-phase foam, liquid content in the foam, and foam stability were measured using the model of the DFA-100 dynamic foam analyzer (KRÜSS, Hamburg, Germany). Firstly, 50 mL of solution prepared according to the experimental conditions is added to the observation column. Then, the observation column is placed on the observation platform, and foam is generated using the inflation method controlled by the computer-operated instrument to record experimental data. Each experimental condition is measured five times, and the average of the five measurements is taken as the final result. The main structure of the testing instrument is shown in Figure 13.

#### 3.3.4. Surface Tension Measurements

Surface tension was measured using the JK-99 surface tension meter (Shanghai Zhongchen Digital Technology Equipment Co., Ltd., Shanghai, China) employing the platinum plate method. Each sample was measured three times, and the average value was taken as the surface tension measurement result.

## 4. Conclusions

Combining collectors can yield better or similar results compared to DDA. Combining OCT or CTAB with DDA can result in higher quartz recovery, while SDS with DDA leads to slightly lower recovery. Among these combinations, CTAB with DDA exhibits the most robust foaming performance, followed by OCT with DDA, whereas SDS with DDA performs weakest. The alteration in CTAB molar content exerts minimal influence on the foaming capacity of the composite collector, whereas SDS exhibits the most significant impact.

Combining SDS or OCT with DDA can decrease the generated foam stability, while CTAB increases it. The influence of the molar content of OCT on the half-life of generated foam is minimal, while CTAB shows the most significant impact. As the molar content of surfactants in combined collectors increases, the induction time between bubbles and particles initially decreases and then increases. A negative correlation exists between induction time and recovery. Additionally, as mineral particle size decreases, induction time decreases and then increases. At the testing condition, lower surface tension generates more bubbles, more uniform bubble size distribution, weaker foam liquid film drainage, more robust foam stability, a larger foam volume, and a higher recovery rate.

## Figures and Tables

**Table 1:** Statistical values of the number of bubbles and the projected area of bubbles for foam generated by different systems.

Collector System	Number of Bubbles per Square Millimeter	$\mathrm{Average}\;\mathrm{Bubble}\;\mathrm{Area}\;\bar{BA}$/μm^2^	$\mathrm{Minimum}\;\mathrm{Bubble}\;\mathrm{Area}\;\bar{{BA}_{min}}$/μm^2^	$\mathrm{Maximum}\;\mathrm{Bubble}\;\mathrm{Area}\;\bar{{BA}_{max}}$/μm^2^
DDA	18.643	53,639	316	12,830,746
OCT/DDA	35.566	28,116	316	1,225,583
CTAB/DDA	44.685	22,379	316	695,032

**Table 2:** Results of chemical multi-element analysis of sample (mass %).

Component	SiO_2_	Al_2_O_3_	K_2_O	CaO	TFe
Content	99.57%	0.3019%	0.0475%	0.0382%	<0.05%

## Data Availability

The data presented in this study are available on request from the corresponding author.
